# Muscarinic and Nicotinic Modulation of Thalamo-Prefrontal Cortex Synaptic Pasticity *In Vivo*

**DOI:** 10.1371/journal.pone.0047484

**Published:** 2012-10-30

**Authors:** Lezio Soares Bueno-Junior, Cleiton Lopes-Aguiar, Rafael Naime Ruggiero, Rodrigo Neves Romcy-Pereira, João Pereira Leite

**Affiliations:** 1 Department of Neuroscience and Behavioral Sciences, Ribeirão Preto School of Medicine, University of São Paulo, Ribeirão Preto, São Paulo, Brazil; 2 Brain Institute, Federal University of Rio Grande do Norte, Natal, Rio Grande do Norte, Brazil; Virginia Commonwealth University, United States of America

## Abstract

The mediodorsal nucleus of the thalamus (MD) is a rich source of afferents to the medial prefrontal cortex (mPFC). Dysfunctions in the thalamo-prefrontal connections can impair networks implicated in working memory, some of which are affected in Alzheimer disease and schizophrenia. Considering the importance of the cholinergic system to cortical functioning, our study aimed to investigate the effects of global cholinergic activation of the brain on MD-mPFC synaptic plasticity by measuring the dynamics of long-term potentiation (LTP) and depression (LTD) *in vivo*. Therefore, rats received intraventricular injections either of the muscarinic agonist pilocarpine (PILO; 40 nmol/µL), the nicotinic agonist nicotine (NIC; 320 nmol/µL), or vehicle. The injections were administered prior to either thalamic high-frequency (HFS) or low-frequency stimulation (LFS). Test pulses were applied to MD for 30 min during baseline and 240 min after HFS or LFS, while field postsynaptic potentials were recorded in the mPFC. The transient oscillatory effects of PILO and NIC were monitored through recording of thalamic and cortical local field potentials. Our results show that HFS did not affect mPFC responses in vehicle-injected rats, but induced a delayed-onset LTP with distinct effects when applied following PILO or NIC. Conversely, LFS induced a stable LTD in control subjects, but was unable to induce LTD when applied after PILO or NIC. Taken together, our findings show distinct modulatory effects of each cholinergic brain activation on MD-mPFC plasticity following HFS and LFS. The LTP-inducing action and long-lasting suppression of cortical LTD induced by PILO and NIC might implicate differential modulation of thalamo-prefrontal functions under low and high input drive.

## Introduction

In the prefrontal cortex (PFC), the rescaling of synaptic weights mediated by long-term potentiation (LTP) and long-term depression (LTD) is thought to play an important role in working memory, decision-making, behavioral inhibition and attention shifting [1]–[3]. Much of the LTP/LTD dynamics in the PFC takes place at afferent terminals from subcortical structures, including the basolateral amygdala, ventral tegmental area, CA1 of the hippocampus, and medial thalamic nuclei [4]. In particular, the mediodorsal thalamic nucleus (MD) is one of the most prominent sources of excitatory projections to the PFC, both in primates and rodents [5]–[10].

Several studies, ranging from functional imaging of the human brain to behavioral tests in animal models, have demonstrated the involvement of MD-PFC reciprocal projections in cognitive functions [11]–[16] and in pathological conditions, especially schizophrenia [17]–[20]. Electrophysiological studies in rodents have also shown that changes in PFC responses mediated by MD stimulation are involved in the modulation of hippocampus-evoked activity in the PFC [21], fear extinction [22], [23], and propagation of limbic seizures [24]–[27].

The MD-PFC circuit can be influenced by ascending cholinergic projections from the brainstem and basal forebrain [28]–[30], which represent important modulators of cognitive processes [31]–[34] and oscillatory activity throughout the sleep-wake cycle [35]–[37]. Unbalanced cholinergic neurotransmission is associated with cognitive decline, schizophrenia, Alzheimer's disease, and temporal lobe epilepsy [38]–[43]. In addition, several studies have shown that cholinergic activation regulates synaptic plasticity in adult thalamocortical loops comprising sensorial areas of the cortex [44]–[48]. However, the cholinergic modulation of thalamus-induced plasticity in associative cortical areas is still poorly understood. In one of the few studies *in vivo* [49], it was shown that nicotinic agonists into the medial prefrontal cortex of rats (mPFC, prelimbic area) facilitated MD-evoked spikes and increased glutamate levels in the mPFC. However, the authors did not evaluate long-term synaptic plasticity in the MD-mPFC pathway. Synaptic plasticity in this pathway was also shown to occur associated with fear learning in mice in the absence of any pharmacological treatment [22], [23].

Recently, we have shown that muscarinic activation of the brain, by an M1 preferential agonist, enhances the hippocampal-mPFC plasticity in two different ways. It specifically potentiates the late-phase LTP induced by high frequency stimulation [50], and promotes a long-lasting LTD in the mPFC induced by trains of low frequency stimulation [51]. Therefore, considering (1) that CA1 and MD project and influence a common set of neurons in the mPFC [52], [53], suggesting a possible substrate for the local interaction between hippocampal inputs and thalamocortical activity; and (2) that these projections can be modulated during general states of cholinergic activation achieved by the administration of muscarinic and nicotinic agonists, we decided to further investigate the muscarinic and nicotinic effects on LTP and LTD in the MD-mPFC circuit *in vivo*.

## Materials and Methods

### 2.1. Subjects

A total of 71 adult male Wistar rats (250–450 g) were housed in standard rodent cages in a colony room maintained at 24°C under a 12 h light/12 h dark cycle with free access to food and water. All procedures were performed according to the Brazilian Council for Animal Experimentation (CONCEA) guidelines and approved by the Ethics Committee of the Ribeirão Preto School of Medicine (protocol number 125/2008). These guidelines abide by the National Institutes of Health rules for the care and use of laboratory animals (NIH Publications No. 8023, revised 1978). Experiments were designed to minimize the number of animals used and their suffering.

### 2.2. Surgery and electrophysiology

Rats were anesthetized with urethane (1.2–1.5 mg/kg, i.p., in NaCl 0.15 M; Sigma-Aldrich, USA) and placed in a stereotaxic frame (David Kopf Instruments, USA), and their body temperature was maintained at 37±0.5°C by using a heating pad (Insight Ltda, Brazil). When necessary, the level of anesthesia was maintained with supplementary injections of the anesthetic (10% of the initial dose) after checking the tail pinch reflex. For electrode and cannula implantation, the skull was exposed and small holes were drilled to allow access to the left hemisphere prelimbic area (PrL) of the mPFC (antero-posterior, AP: +3.0 mm; lateral to midline, L: −0.4 mm; ventral to dura mater, V: −3.2 mm), left hemisphere MD (AP: −1.9 mm, L: −0.4 mm, V: −4.8 mm) and right hemisphere lateral ventricle (LV; AP: −0.5 mm, L: +1.3 mm, V: −2.5 mm) according to the rat brain atlas [54]. An additional hole was drilled over the parietal cortex in the right hemisphere to implant a micro-screw used as recording reference. Thereafter, a 23-gauge stainless-steel cannula was inserted into the brain and positioned 1 mm above the LV. The cannula was fixed to the skull with dental acrylic resin.

Teflon-insulated tungsten wires (60 µm diameter) were used to prepare stimulating and recording electrodes. A twisted bipolar electrode (vertical tip separation: 500 µm) was used for constant current stimulation of the MD and a monopolar electrode was used to record field post-synaptic potentials (fPSPs) in the mPFC. Both electrodes were lowered into the brain through the holes drilled on the skull, after removing the dura mater. Monophasic test pulses (200 µs duration, 150–200 µA; S88 Stimulator, Grass Technologies, USA) were delivered through the bipolar electrode every 20 s, and the final position of the electrodes was adjusted to obtain the highest negative-going fPSP in the mPFC (amplitude ≥150 µV). fPSPs were amplified and filtered (×100, 0.01–1 KHz; P55-AC Pre-amplifier, Grass Technologies, USA) before digitization at 10 KHz (PowerLab/16S; ADInstruments, Australia). For some animals, it was necessary to invert the polarity of the stimulation prior to the beginning of the experiments in order to increase the regularity of the fPSP. Although polarity influenced direction of stimulus artifact, it did not affect the latencies of fPSP negative peaks. Once the electrodes were positioned and the stimulation polarity was defined, electrical pulses were delivered every 20 s at increasing intensities (60–500 µA) and the fPSP amplitudes were used to calculate input-output curves for each animal. Based on the input-output curves, we obtained the intensity necessary to produce maximum fPSP amplitudes and used 60–70% of such intensity to stimulate the MD during baseline, LTP or LTD induction and post-LTP or LTD recordings.

Baseline fPSPs were recorded for 30 min with single electrical pulses (200 µs duration; every 20 s). Then, the drugs were microinjected through a 30-gauge needle inserted into the cannula and connected to a 10 µL microsyringe (Hamilton Company, USA) via a polyethylene tube. After microinjection, LTP or LTD was induced by delivery ofhigh-frequency (HFS) or low-frequency (LFS) trains of stimuli into the MD, respectively. Post-HFS/LFS recordings of fPSPs resumed for an additional 240 min to monitor the dynamics of mPFC responses. The HFS protocol consisted of two series (10 min apart) of 10 trains of 50 pulses (250 Hz). These trains were delivered every 10 s [23], [50], [55]. LFS consisted of a single train of 1200 pulses (2 Hz) [23].

### 2.3. Cholinergic drugs

We used the following drugs: (1) pilocarpine hydrochloride (PILO, Sigma-Aldrich, USA), a non-selective muscarinic agonist with high affinity for M1-like receptors [56], [57]; and (2) (-)-nicotine hydrogen tartrate (NIC, Sigma-Aldrich, USA), an agonist with high affinity for neural nicotinic receptors, especially α7 and α4β2 subtypes [58], [59]. Artificial cerebrospinal fluid (aCSF; in mM: 2.7 KCl, 1.2 CaCl_2_, 1.0 MgCl_2_, and 135.0 NaCl, with pH 7.3 at room temperature) was used to dissolve both PILO (40 nmol/µL) and NIC (320 nmol/µL) salts. The concentrations of PILO and NIC were chosen based on a pilot experiment that measured the duration of the oscillatory changes induced in the mPFC and MD, and did not produce alteration of the physiological parameters of the animals, such as heart rate and salivation. aCSF without PILO or NIC was used as the control vehicle. The injections of PILO, NIC, or aCSF were delivered by intracerebroventricular route (i.c.v) in a volume of 1 µL over a two-minute period.

### 2.4. Experimental design

To investigate the cholinergic modulation of MD-evoked synaptic plasticity in the mPFC, three experiments were carried out. Experiment I tested the effects of cholinergic modulation on the induction and maintenance of LTP. For that, animals received PILO, NIC, or aCSF immediately before HFS and were divided into three groups: PILO-HFS, NIC-HFS, and aCSF-HFS, respectively. Experiment II tested the effects of cholinergic modulation on the induction and maintenance of LTD. Animals received PILO, NIC, or aCSF just before LFS and were also divided into three groups: PILO-LFS, NIC-LFS, and aCSF-LFS, respectively. Experiment III assessed the effects of PILO, NIC, or aCSF on basal mPFC responses induced by MD stimulation. Animals received PILO, NIC, or aCSF, but did not receive train stimulation and were grouped as PILO-Ctrl, NIC-Ctrl, and aCSF-Ctrl. Synaptic plasticity was analyzed by quantifying the average fPSP amplitude normalized to the baseline at different time points after synaptic plasticity induction. For that, fPSP amplitudes were averaged every 10 min and normalized as percentage of the baseline mean amplitude.

### 2.5. Local field potential analysis

To monitor the state of brain activity associated to the muscarinic (PILO) and nicotinic (NIC) modulation, we recorded local field potentials (LFP) simultaneously in the MD and mPFC through the same electrodes used to induce and record LTP or LTD. Thalamic and neocortical LFPs were recorded during a 6 min period divided into 2 min blocks: before, during and after i.c.v microinjection. After down sampling to 200 Hz and low-pass filtering (0.5–100 Hz), Welch's power spectral densities (Hanning window) were calculated every 10 s epochs. Spectral densities were estimated for each epoch after averaging periodograms calculated from eight sections with 50% overlap. Delta (0.5–4 Hz), theta (4–12 Hz), beta (12–30 Hz) and gamma (30–80 Hz, removing 58–62 Hz noise) normalized powers were calculated using custom-made MATLAB scripts (The MathWorks, Natick, MA). Normalized band powers were compared to evaluate the effects of PILO and NIC on the oscillatory activity recorded in the MD and mPFC.

### 2.6. Histology

After each recording session, a current pulse (1 mA, 1 s) was delivered through the stimulation and recording electrodes to produce a small electrolytic lesion for electrode localization. The animals received an additional injection of the anesthetic and had their brains removed after decapitation. The brains were post-fixed in 10% formaldehyde-saline solution for 14 h at 4°C and cryoprotected for 48 h in 20% sucrose solution (in 0.1 M sodium phosphate buffer, pH 7.4). After rapid freezing in dry ice-chilled isopentane, 30 µm-thick slices were cut in a cryostat, mounted on gelatinized slides and stained with cresyl violet. Electrode tip positions and cannula tracts were determined after analysis of brain sections under the optic microscope (AxioPhot, Carl Zeiss Inc.).

### 2.7. Statistical analysis

Analysis of group differences following HFS or LFS was carried out by two-way ANOVA with repeated measures (group: fixed factor vs. time: repeated measures). The same ANOVA was used to test power spectrum differences in the mPFC and MD along the 6 min recording of LFPs. The Newman-Keuls *post hoc* test was applied following ANOVAs when necessary. All results are expressed as the mean ± SEM and significance level was set to 0.05.

## Results

### 3.1. Accuracy of implants

**Figure 1:**
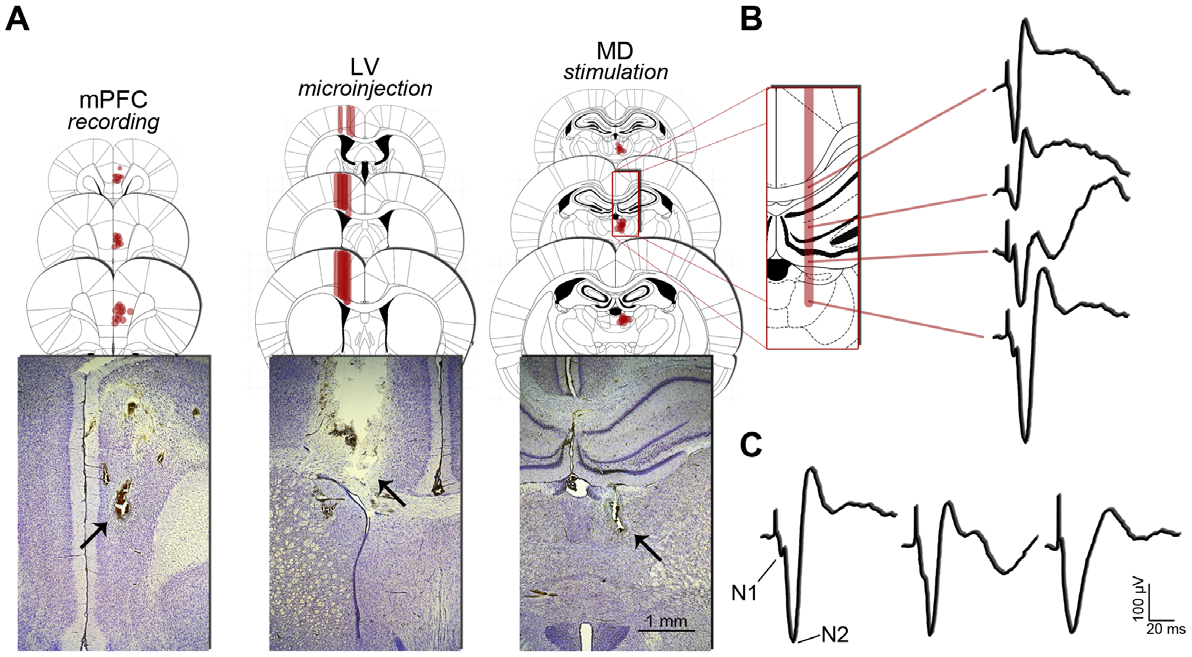
Histological validation of implants and typical prefrontal fPSPs. (A) Positioning of electrodes and cannulae from a coronal point of view. For mPFC and MD, coronal plates represent the anteroposterior variation of the electrode tip positioning (red dots), preferentially at the PrL of mPFC (layer-inespecific) and the anterior half of MD (subdivision-inespecific), both in the left hemisphere. For LV, the coronal plates show the variation of the cannula positioning (red bars) just above the right lateral ventricle, where the experimental drugs were injected. In the representative Nissl-stained coronal sections, the arrows point to typical electrolytic lesions (applied after the end of the experiments) and cannula tract. (B) Once the recording electrode was positioned at the mPFC, a typical dorsoventral profile of fPSPs was consistently evoked across subjects, while the stimulation electrode was lowered towards the MD (see details in the text). (C) Diversity of MD-evoked fPSPs recorded in the mPFC. The first fPSP shows a clear differentiation between two distinct negative peaks, which we termed N1 (amplitude 108.20±9.32 µV; latency 6.85±0.15 ms) and N2 (amplitude 270.00±17.10 µV; latency 13.43±0.17 ms). Such an aspect of fPSP was obtained in approximately half the subjects. In some cases, like the second fPSP, the N1 peak was subtle. Finally, in some other cases, like the third fPSP, the N1 peak was indistinguishable.

All animals included in our analysis had the stimulation electrode tips positioned within the MD, most often in its anterior and medial aspects, which contain the highest density of mPFC-projecting cells according to retrograde tracing [60]. Recording electrode tips were most frequently observed in the medial wall of the ipsilateral mPFC, at the level of PrL. Cannulae placement was observed approximately 1 mm above the LV, so that only the microinjection needle reached the LV (Figure 1).

### 3.2. Characteristics of mPFC responses evoked by MD stimulation

In all experiments, we first positioned the recording electrode at the PrL area of the mPFC based on stereotaxic coordinates [54] and then, lowered the stimulation electrode in 200 µm steps while applying pulses each 20 s (electrical parameters described in the methods section). No fPSP was found until the stimulation electrode reached 3.0 mm below the dura mater (presumably at the corpus callosum level), from where we consistently elicited fPSPs with a negative peak at latency of ∼9 ms (Figure 1). As the stimulation electrode crossed the hippocampus on its way towards the MD, the same profile of fPSP was repeatedly elicited until approximately 4.2–4.4 mm below the dura mater, when we observed a shift of the negative peak from a latency of ∼9 to ∼13 ms. From that point on, we continued to lower the electrode until 4.8–5.2 mm below dura mater (at the MD level), when we obtained the strongest and most reliable negative-going fPSP and concluded the implantation. The prefrontal fPSPs obtained by stimulation throughout the hippocampus may occur due to the activation of passing fibers, since retrograde tracing data from the literature do not show hippocampal cells projecting to the mPFC at the anteroposterior level where our stimulation electrodes were implanted [59]. Nevertheless, fPSPs elicited during the trajectory of the stimulating electrode were useful as references for the refinement of the dorso-ventral implant position.

In approximately half of the subjects, the MD-evoked fPSPs showed two distinct negative peaks, which we termed N1 and N2 (Figure 1). When clearly detected, N1 was a low-amplitude (108.20±9.32 µV) short-latency (6.85±0.15 ms) negative peak, but in some cases it was too subtle to be defined. Differently, N2 was a negative peak characterized by high amplitude (270.00±17.10 µV) and long latency (13.43±0.17 ms), and was consistently detected in all subjects. fPSPs recorded in the present study had latency and amplitude profiles resembling those previously described in awake mice [22], [23]. As reported by these authors, it was difficult to dissociate the short-latency component of the fPSP depending on the case. Thus, we adopted a similar definition and used measurements of the consistent N2 peak amplitude as an index of field synaptic response in the mPFC. Table 1 shows baseline N2 parameters for all groups in the present study. As expected, no significant differences were detected between groups when latency and amplitude were analyzed (one-way ANOVA; *p*>0.05).

**Table 1:** Amplitude and latency of MD-evoked fPSPs recorded in the mPFC during baseline.

Groups	Amplitude (µV)	Latency (ms)
PILO-HFS	231.25±31.19	13.22±0.31
NIC-HFS	250.00±56.78	14.57±0.39
aCSF-HFS	335.00±83.00	13.41±0.42
PILO-LFS	277.50±31.83	13.22±0.51
NIC-LFS	262.50±64.96	13.65±0.68
aCSF-LFS	341.25±63.82	13.16±0.36
PILO-Ctrl	255.00±31.40	11.99±0.44
NIC-Ctrl	285.00±50.92	14.01±0.60
aCSF-Ctrl	190.00±14.52	13.72±0.57

Intergroup one-way ANOVA showed no significant differences. Data are shown as the mean ± SEM.

Pirot et al. [5], [61] showed that MD stimulation evoked two categories of unitary responses in the mPFC, which were distinguished by their latencies: short (3.46±0.05 ms) and long (13.67±0.22 ms). Short-latency responses correspond to the actual recruitment of MD-mPFC thalamocortical fibers, whereas long-latency responses correspond to the activation of intracortical axon collaterals, originating from mPFC-MD corticothalamic fibers. Indeed, electrical pulses applied within the MD inevitably stimulate mPFC-derived axon terminals, eliciting antidromic action potentials towards the mPFC, and thereby recruiting the axon collaterals of corticothalamic fibers [6]. Differently from Pirot et al. [5], [61], Herry et al. [22] were the first to examine MD-evoked fPSPs in the mPFC to study long-term synaptic plasticity in the thalamocortical circuit, interpreting the short-latency component of their fPSPs as a response to MD-mPFC activation. However, given that the they were not always able to identify the short-latency component depending on the subject, the authors (as well as Herry and Garcia [23]) measured the amplitude of the long-latency component (N2) of the fPSPs. Similarly, we decided to use N2 as an index of mPFC plasticity. Despite the low selectivity of MD electrical stimulation, we consider that MD-evoked plasticity may control the excitatory reverberation in the MD-mPFC circuit as a whole. In addition, Herry et al. [22] and Herry and Garcia [23] showed that LTD of MD-evoked fPSPs correlate to learning behaviors (i.e., resistance to extinction of conditioned fear), reinforcing the functional implications of MD-mPFC long-term plasticity.

### 3.3. Concentration-dependent effects of PILO and NIC on MD and mPFC oscillations

**Figure 2:**
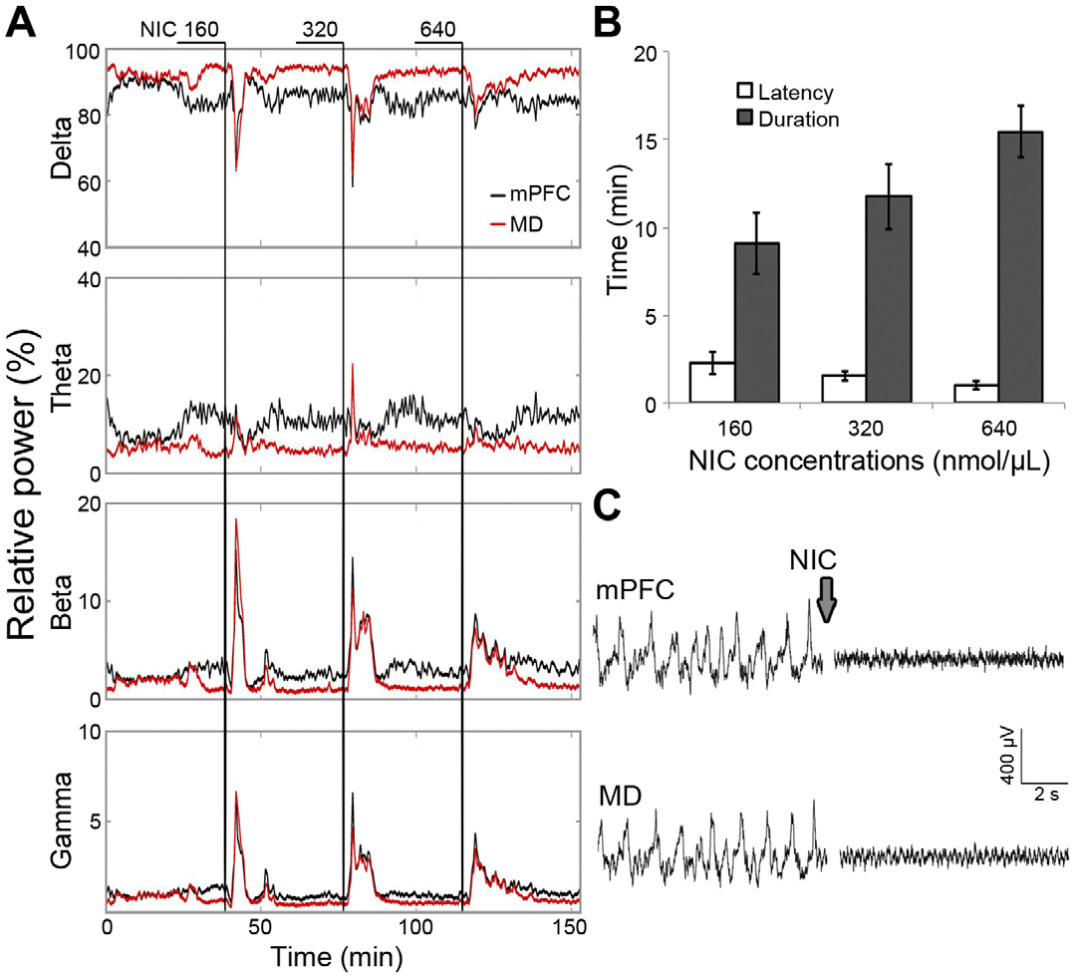
Concentration-dependent effect of NIC on forebrain oscillatory activity. Different concentrations of NIC (160, 320, and 640 nmol/µL; 1 µL icv) were injected while LFPs were continuously recorded during 120 min for analysis of the power spectrum at delta (0.5–4 Hz), theta (4–12 Hz), beta (12–30 Hz), and gamma (30–80 Hz) frequency bands. (A) Continuous thalamic and cortical LFP recording from a representative subject. (B) Analysis of latency and duration of LFP changes induced by the injection of the different NIC concentrations in a sample of eight rats. The sequence of injections at the different concentrations was randomized (data shown as the mean ± SEM). (C) Representative EEG tracings from mPFC and MD before and after NIC injection. Based on these experiments, we decided to use NIC 320 nmol/µL to induce a transient effect matching the duration of HFS and LFS protocols.

We have recently determined the latency and duration of the muscarinic effect of PILO on LFP oscillations in the hippocampus and mPFC following i.c.v. injections of different concentrations of the drug. PILO 40 nmol/µL (injected volume of 1 µL) shifts the pattern of urethane-driven slow waves to a transient state of increased high-frequency oscillations for ∼15 min with a latency ∼1 min. In the present study, we show that LFPs recorded in the MD and mPFC are also shifted towards faster oscillations in a concentration-dependent manner in response to NIC (Figure 2). In particular, the effect of NIC 320 nmol/µL (1 µL, i.c.v.) on thalamic and cortical LFPs lasted for ∼12 min, matching the duration of HFS and LFS protocols used in this study. Therefore, PILO and NIC were used at these concentrations (respectively, 40 nmol/µL and 320 nmol/µL) in all experiments.

### 3.4. Cholinergic modulation of the oscillatory activity in the hippocampus and mPFC

**Figure 3:**
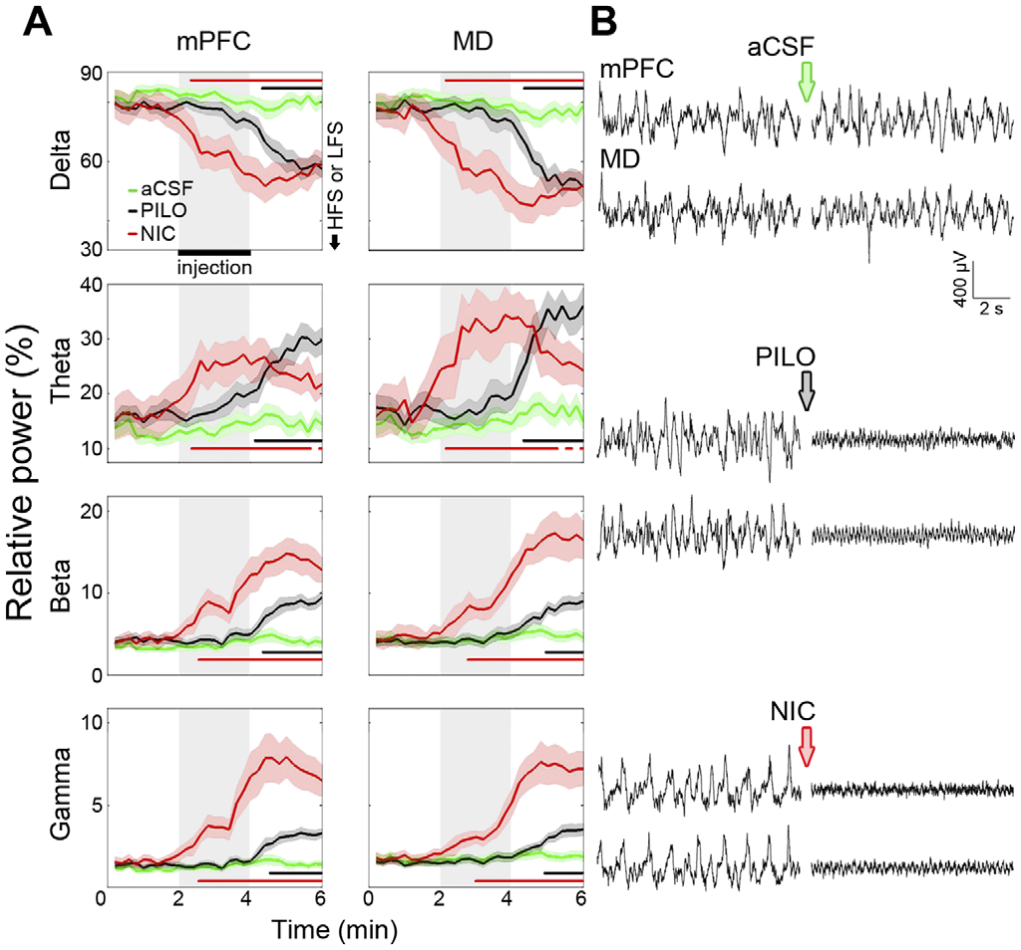
LFP power spectrum comparing mPFC and MD oscillatory activity before, during, and after microinjection. (A) Charts detailing PILO and NIC effects on LFPs, showing a decrease in delta (0.5–4 Hz), as well as an increase in theta (4–12 Hz), beta (12–30 Hz), and gamma (30–80 Hz) relative power. The LFP changes induced by NIC occurred earlier than those induced by PILO, with a shorter duration of theta potentiation, and a stronger potentiation of beta and gamma waves. The data were obtained from all aCSF, PILO and NIC rats of the synaptic plasticity experiments. Significant differences are indicated by two-way repeated measures ANOVA followed by the Newman-Keuls post-hoc test (black bar: aCSF vs. PILO; red bar: aCSF vs. NIC). (B) Representative EEG tracings from mPFC and MD before and after icv microinjections. Data are shown as the mean ± SEM.

We quantified the spectral content of cortical and thalamic LFPs before, during, and after PILO and NIC microinjections. Figure 3 shows the integrated relative power spectra changes in MD and mPFC LFPs along the 6 min period of LFP continuous recording. We can see that PILO and NIC significantly decreased delta and proportionally potentiated theta, beta, and gamma oscillations. Particularly, the NIC effects on the four frequency bands had shorter latencies than the PILO effects, since the latter were already evident during the microinjection window. In addition, NIC induced a stronger potentiating effect on beta and gamma. Microinjection of aCSF by itself did not alter the urethane-induced slow-wave context. The ANOVA *F* values for interaction effects are as follows. mPFC delta: *F*_(58,1653)_ = 5.181; mPFC theta *F*_(58,1653)_ = 4.039; mPFC beta: *F*_(58,1653)_ = 8.930; mPFC gamma: *F*_(58,1653)_ = 13.880; MD delta: *F*_(58,1653)_ = 6.040; MD theta: *F*_(58,1653)_ = 5.538; MD beta: *F*_(58,1653)_ = 13.643; MD gamma: *F*_(58,1653)_ = 16.578. The *p* values for all the ANOVAs were less than 0.001.

### 3.5. Cholinergic activation triggers a delayed form of LTP in the mPFC

**Figure 4:**
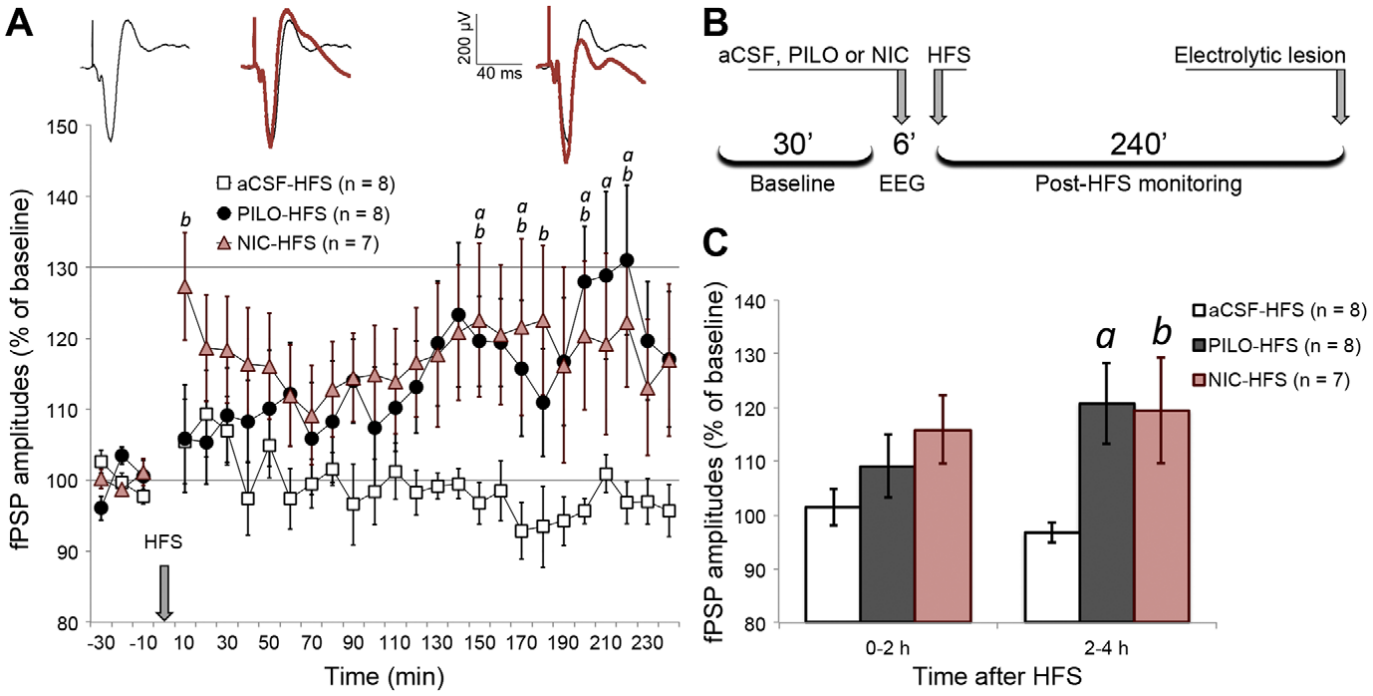
HFS induced a late LTP in mPFC only when applied under PILO and NIC effects. (A) fPSP amplitude throughout baseline (30 min) and post-HFS monitoring (240 min), depicting amplitudes averaged in 10-min blocks and normalized in relation to baseline mean amplitude. Significant differences are indicated by two-way ANOVA with repeated measures, followed by the Newman-Keuls post-hoc test (a = PILO vs. aCSF; b = NIC vs. aCSF; p<0.05). The sequence of averaged fPSPs above the chart represents a typical PILO-HFS experiment, where post-HFS fPSPs (red) are superimposed on baseline fPSPs (black). Such fPSPs are roughly aligned with the time course of the chart. (B) Timeline summarizing the procedures for HFS experiments. (C) Data from chart A clustered in blocks of 2 h after HFS, highlighting PILO and NIC significant effects restricted to the second half of the monitoring. Data are shown as the mean ± SEM.

Application of HFS in the MD did not induce LTP in the mPFC by itself. The amplitude of mPFC fPSPs recorded following aCSF injection (aCSF-HFS group) did not change for 4 h (Figure 4). In contrast, we observed that both PILO and NIC induced a delayed-onset form of LTP, with similar kinetics (PILO-HFS and NIC-HFS groups; interaction effect: *F*_(46,460)_ = 1.714; *p* = 0.003). As depicted in figure 4, LTP induced by PILO and by NIC began to emerge approximately 2 h after HFS, with values reaching approximately 120–130% of baseline level.

### 3.6. Cholinergic activation suppresses a long-lasting form of LTD in the mPFC

**Figure 5:**
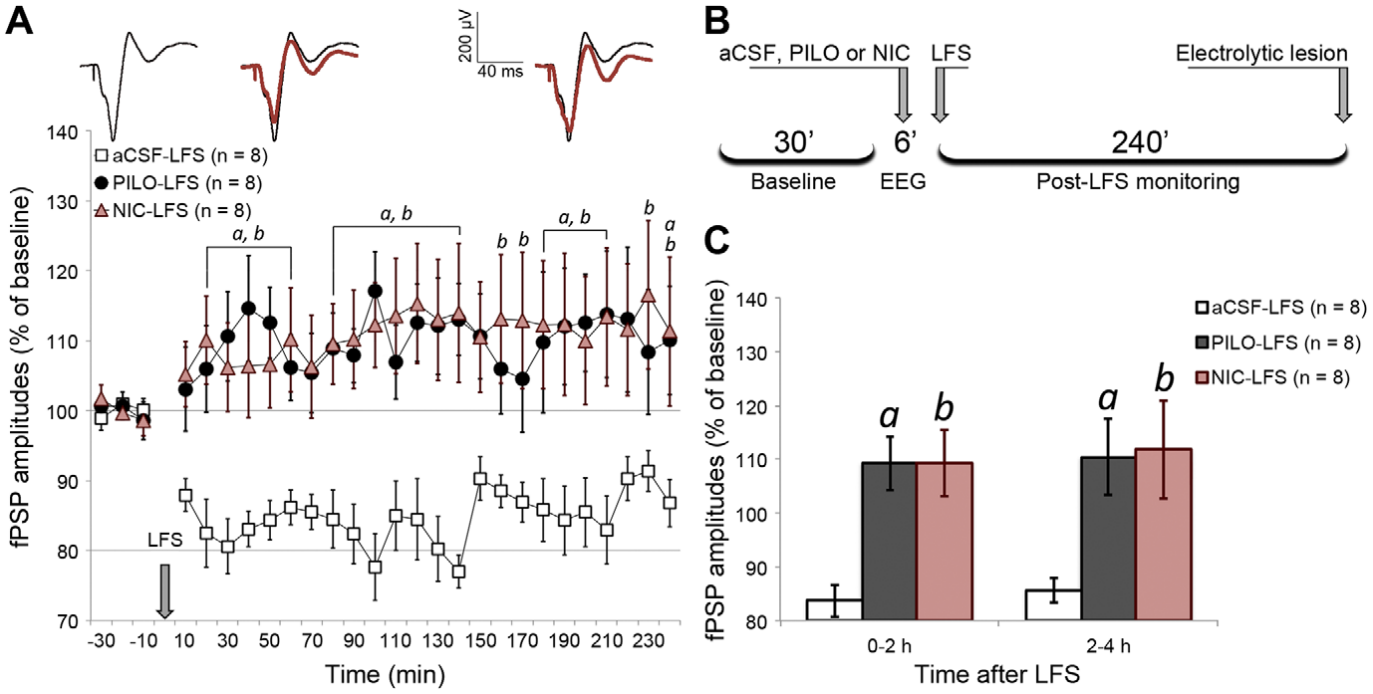
LFS induced a stable LTD in mPFC only when applied under urethane-driven slow-wave context. (A) fPSP amplitude throughout baseline (30 min) and post-LFS monitoring (240 min), depicting amplitudes averaged in 10 min blocks and normalized in relation to baseline mean amplitude. Significant differences are indicated by two-way ANOVA with repeated measures, followed by the Newman-Keuls post-hoc test (a = PILO vs. aCSF; b = NIC vs. aCSF; p<0.05). The sequence of averaged fPSPs above the chart represents a typical aCSF-LFS experiment, where post-LFS fPSPs (red) are superimposed on baseline fPSPs (black). Such fPSPs are roughly aligned with the time course of the chart. (B) Timeline summarizing the procedures for LFS experiments. (C) Data from chart A clustered in blocks of 2 h after LFS, showing the stability of PILO and NIC effects throughout the monitoring. Data are shown as mean ± SEM.

**Figure 6:**
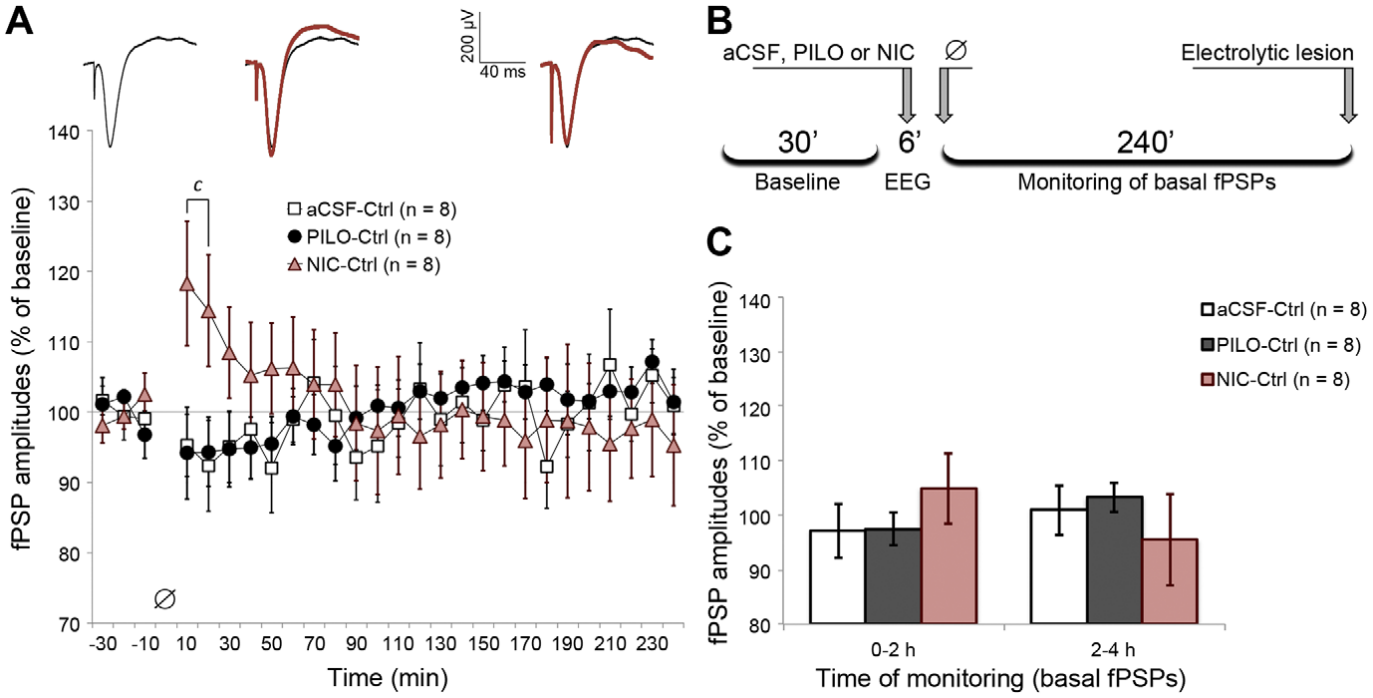
PILO and NIC microinjection alone did not induce long-term changes in MD-evoked prefrontal fPSPs. (A) fPSP amplitude throughout baseline (30 min) and monitoring (240 min), depicting amplitudes averaged in 10 min blocks and normalized in relation to baseline mean amplitude. Significant differences are indicated by two-way ANOVA with repeated measures, followed by the Newman-Keuls post-hoc test (c = NIC vs. both aCSF and PILO; p<0.05). The sequence of averaged fPSPs above the chart represents a typical NIC-Ctrl experiment, where fPSPs recorded during the 4 h monitoring (red) are superimposed on baseline fPSPs (black). Such fPSPs are roughly aligned with the time course of the chart. (B) Timeline summarizing the procedures for Ctrl experiments, in which the empty-set symbol represents absence of train stimulation. (C) Data from chart A clustered in blocks of 2 h of monitoring. Data are shown as the mean ± SEM.

In the groups in which LFS was applied after aCSF microinjection, we observed stable LTD with duration of 4 h, at 80–90% of baseline level (Figure 5). In contrast, PILO-LFS and NIC-LFS subjects showed a complete suppression of LTD throughout the 4 h monitoring period. The effects had similar kinetics for both groups and apparently converted the LTD into a subtle, but stable LTP (Figure 6; group effect: *F*_(2,21)_ = 6.719; *p* = 0.006).

### 3.7. Basal mPFC fPSPs are not affected by NIC or PILO in the long term

While PILO and NIC modulated HFS and LFS effects on MD-evoked prefrontal responses, these agonists alone did not affect mPFC responses in the long term. Although we observed a brief potentiation induced by NIC in the first 20 min (interaction effect: *F*_(46,480)_ = 2.148; *p*<0.001), mPFC responses quickly recovered and did not show the sustained effect observed after HFS and LFS (Figure 6). These results support the fact that the cholinergic modulation observed after HFS and LFS is due to the interaction between the stimulation protocols and the cholinomimetic states promoted by PILO and NIC.

By separately comparing aCSF-LFS with aCSF-Ctrl or aCSF-HFS curves, we showed that LTD was significantly induced after LFS, but our HFS protocol was not sufficient to induce LTP (aCSF-LFS vs. aCSF-Ctrl: group effect, *F*_(1,14)_ = 7.638, *p* = 0.015, and time effect, *F*_(23,315)_ = 1.587, *p* = 0.045; aCSF-LFS vs. aCSF-HFS: group effect, *F*_(1,14)_ = 19.074, *p*<0.001, and interaction effect, *F*_(23,315)_ = 1.734, *p*<0.021; two-way repeated measures ANOVA; Figure S1). Given the subtle and stable LTP (∼110%) observed in PILO-LFS and NIC-LFS groups, four additional comparisons were made: PILO-LFS vs. PILO-Ctrl, PILO-LFS vs. PILO-HFS, NIC-LFS vs. NIC-Ctrl, and NIC-LFS vs. NIC-HFS. All comparisons showed no intergroup differences. Thus, PILO and NIC were able to induce LTP (up to ∼130%, Figure 4) when applied before an ineffective HFS protocol, whereas they promoted a stable but of lower magnitude LTP following the LTD-inducing LFS protocol.

### 3.8. Thalamic and cortical oscillatory activity correlates with the peak of prefrontal LTP

To test whether the magnitude of prefrontal fPSPs correlated to the level of oscillatory changes induced by PILO and NIC in the MD and mPFC, we pooled data from all rats used in the synaptic plasticity experiments and divided them into three major groups (HFS, LFS, and Ctrl), regardless the injection they received. We then calculated the Pearson's linear correlation between the relative power at four frequency bands (delta, theta, beta, and gamma) and the mean fPSP amplitude every10 min blocks following HFS, LFS, or Ctrl.

**Figure 7:**
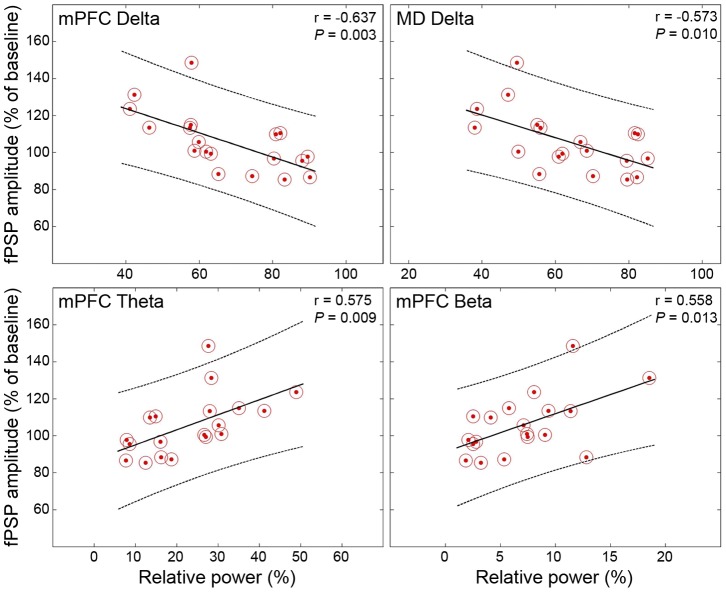
There were correlations between the level of LFP changes and fPSP amplitudes throughout the monitoring. The four plots represent the highest concentration of significant correlations, specifically between the delta, theta, and beta bands recorded prior to HFS and the 120–150 min time point after HFS. The lower the delta in mPFC and MD, the higher the fPSP amplitudes (top). The higher the theta-beta in mPFC, the higher the fPSP amplitudes (bottom).

In HFS rats, the most evident results show a negative correlation between the amplitude of cortical fPSPs and the relative power of delta recorded in the mPFC and MD throughout the experiments. In addition, we observed a positive correlation of the amplitude of cortical fPSPs with the relative power of theta and beta recorded specifically in the mPFC, Therefore, the lower the relative delta power after microinjection, the higher the prefrontal responses to MD stimulation during the 4 h monitoring. Similarly, the higher the relative theta and beta powers after microinjection, the higher the prefrontal responses. The significant correlations were particularly concentrated 120 min after HFS, nearly matching the peak of prefrontal LTP in our HFS experiments (Figure 7).

Significant correlations were also found in LFS rats although they were less frequent and occurred only during the first 150 min of monitoring. Significant results were restricted to delta (negative correlations) and theta oscillations (positive correlations) recorded both in the mPFC and MD (e.g., at 20 min after LFS, MD delta: r = −0.497, *p* = 0.026; MD theta: r = 0.489, *p* = 0.028; at 30 min after LFS, mPFC delta: r = −0.521, *p* = 0.038; mPFC theta: r = 0.537, *p* = 0.032). Therefore, delta and theta powers after microinjection were negatively and positively correlated with the prefrontal responses to MD stimulation during the first half of the monitoring respectively, which is consistent with the LTD-suppressing effects of PILO and NIC in our experiments.

## Discussion

The present study describes the effects of the muscarinic and nicotinic brain activation on the long-term synaptic plasticity in the mPFC induced by electrical stimulation of the MD *in vivo*. We can divide our results into five main findings: (1) the muscarinic and nicotinic activation, induced by PILO and NIC, promoted a delayed-onset LTP in the mPFC when applied prior to HFS; (2) in contrast, both PILO and NIC suppressed LTD in the mPFC triggered by LFS; (3) PILO and NIC did not affect basal synaptic transmission in the long term, but NIC showed a transient potentiating effect both in the control and HFS condition with a mean duration of 20 min; (4) the network effects of PILO and NIC were detected by a transient decrease in the prevalence of delta waves (0.5–4 Hz) and a proportional increase of fast oscillations (4–80 Hz) in the cortex and thalamus; and (5) although PILO and NIC induced brief oscillatory changes in the MD and mPFC, such changes showed significant correlation to the increase in fPSP amplitudes recorded more than two hours after HFS or LFS.

In two recent reports, we used a similar design in anesthetized rats to assess the muscarinic modulation of LTP and LTD in the hippocampus-mPFC pathway [50], [51]. We showed that muscarinic activation, produced by systemic administration of PILO prior to HFS in CA1, prevented the decay of LTP 2 h after its induction [50]. In contrast, the intracerebroventricular administration of PILO converted a subthreshold transient synaptic depression into a robust and stable LTD, lasting up to 4 h [51]. These results indicate that the brain muscarinic activation enhances both forms of synaptic plasticity in the mPFC suggesting an important cholinergic role in the bidirectional control of hippocampo-prefrontal plasticity. Our present findings, on the other hand, support a distinct function for the cholinergic modulation of MD-evoked mPFC plasticity, in which both muscarinic and nicotinic agonists either enhance LTP or suppress LTD. In fact, PILO and NIC converted a subthreshold HFS into a late-onset LTP and completely blocked LTD induced by LFS, with a net potentiating effect after HFS and a net suppressive effect after LFS. Besides, in the absence of stimulation, the application of NIC induced a transient enhancement of MD-mPFC responses that decayed to basal levels in 20 min. However, it is still unknown if NIC produces similar effects on the CA1-mPFC responses *in vivo*.

Neurochemically, the reciprocal communication between MD and mPFC is mediated by AMPA and NMDA receptors, and regulated by several neuromodulators [5], [49], [61]–[65]. It is well described that NMDA-dependent LTP and LTD relies on the intracellular signaling mediated by cytosolic Ca^2+^, which controls AMPA receptor trafficking to and from the postsynaptic density [67]. These mechanisms can be triggered by HFS or LFS, and enhanced by simultaneous activation of M1-like muscarinic receptors that are widely distributed in the frontal cortex, resulting in sustained or reduced membrane depolarization [31], [68]–[71]. Moreover, some reports have shown that presynaptic nicotinic receptors, mainly the low-affinity α7 and high-affinity α4β2 subtypes, can exert a calcium-dependent potentiation of the thalamocortical transmission [34], [47], [59], [72], [73], which could explain the net potentiating effects of PILO and NIC. Consistently, Gioanni et al. [49] have shown that nicotinic agonists facilitate MD-evoked firing and promote glutamate release in the mPFC. The authors also observed that unilateral MD lesions reduced in ∼40% the binding of 3H-nicotine in the mPFC, indicating that thalamic presynaptic terminals in the mPFC are rich in nicotinic receptors. However, our data do not allow us to make a clear dissection of the individual effects of PILO and NIC on specific receptor subtypes, as we did not use cholinergic antagonists to block their actions.

Although previous studies have shown that excitability and neurotransmission in thalamocortical loops are susceptible to cholinergic modulation [46], [48], [66], there is still a lack of understanding on how synaptic plasticity in the MD-mPFC is affected by cholinergic-driven brain states. Here, our strategy of injecting PILO or NIC into the ventricle of anesthetized rats allowed us to achieve a global cholinergic activation that tried to mimic a physiological state of arousal or rapid-eye-movement (REM) sleep, which are endogenously regulated by ascending projections from the brainstem, basal forebrain, and septum [29], [30]. Despite the limitation of recording during anesthesia, it was recently shown that urethane anesthesia mimics the state alternations of sleep, suggesting its possible use as a model to study sleep oscillations [95]. Interestingly, the cholinergic control of oscillatory states of sleep is relatively preserved by urethane [90]–[95]. Indeed, the transient effects of PILO and NIC on LFPs under urethane resembled the oscillatory pattern observed in REM sleep episodes [84], which are primarily induced by cholinergic projections depolarizing thalamocortical cells and inducing their tonic firing [36], [85], [88]–[89].

A plausible implication of our findings is that acetylcholine through muscarinic and nicotinic receptors might favor MD inputs to the mPFC during cholinergic-driven states such as in sleep or during cognitive demands during waking, leading to a long lasting strengthening of thalamo-prefrontal communication. More specifically, high-frequency inputs to mPFC during REM sleep might induce LTP in thalamocortical synapses, as shown from the effects of PILO and NIC following HFS. In agreement to that, cortical synaptic plasticity seems to occur in sleep and is thought to be a mechanism of consolidation of memories traces [37], [86], [87]. In contrast, low-frequency trains of MD spikes would more efficiently cause synaptic depression in the mPFC in a low cholinergic activity condition. Such LTD-favoring effect would be congruent with the hypothesis of sleep-dependent synaptic homeostasis, according to which slow-wave activity downscales prefrontal synapses, preparing them for ensuing wakefulness [96]. It is know that cholinergic cells of the basal forebrain and septum are also particularly active during wakefulness and are involved in the phasic and tonic cholinergic discharges during cue-detection tasks [81]. Such neuromodulation is thought to raise the sensitivity of cortical networks to afferent inputs, supporting arousal [33], [46]–[48], [74]–[76] and enhancing the thalamocortical signal-to-noise ratio during cognitive and attention-demanding tasks [77]–[81]. It is possible that cognitive processes requiring mPFC activity, such as working memory and action planning, could undergo a state-dependent bimodal cholinergic modulation [2], [82]–[83].

In conclusion, the present study shows that the brain cholinergic activation by PILO and NIC differentially modulate LTP and LTD in the mPFC driven by the thalamus. Considering that a cholinergic unbalance in limbic circuits connected to the prefrontal cortex may contribute to major disorders, such as Alzheimer's disease and schizophrenia, new studies on network plasticity in freely behaving animals under low and high-cholinergic tone may help to elucidate some of the prefrontal roles in such dysfunctions.

## Supporting Information

Figure S1 **Pairwise comparisons between drug-treated groups (aCSF, PILO and NIC) in all experimental conditions (Control, LFS and HFS).** Normalized amplitude of fPSPs recorded during baseline (30 min) and post-tetanization (240 min) are plotted in 10-min blocks. (A) aCSF; (B) PILO; (C) NIC. Significant differences were evaluated by two-way ANOVA with repeated measures, followed by Newman-Keuls post-hoc test. *, p<0.05. All curves correspond to data shown in figures 4–6. Data are shown as mean ± SEM.
(TIF)
