# Correction: Muscarinic and Nicotinic Modulation of Thalamo-Prefrontal Cortex Synaptic Plasticity *In Vivo*

**DOI:** 10.1371/annotation/d2dbf233-db12-431f-b9b6-4bd31cbca23e

**Published:** 2012-11-08

**Authors:** Lezio Soares Bueno-Junior, Cleiton Lopes-Aguiar, Rafael Naime Ruggiero, Rodrigo Neves Romcy-Pereira, João Pereira Leite

The word "Plasticity" is misspelled in the article title. The correct title is: Muscarinic and Nicotinic Modulation of Thalamo-Prefrontal Cortex Synaptic Plasticity *In Vivo*.

The correct citation is: Bueno-Junior LS, Lopes-Aguiar C, Ruggiero RN, Romcy-Pereira RN, Leite JP (2012) Muscarinic and Nicotinic Modulation of Thalamo-Prefrontal Cortex Synaptic Plasticity *In Vivo*. PLoS ONE 7(10): e47484. doi:10.1371/journal.pone.0047484 

