# Supplementary figures and images for: Muscarinic and Nicotinic Modulation of Thalamo-Prefrontal Cortex Synaptic Pasticity In Vivo
Source: PLoS One. 2012 Oct 30;7(10):e47484. doi: 10.1371/journal.pone.0047484 (PMC3484139; doi:10.1371/journal.pone.0047484)

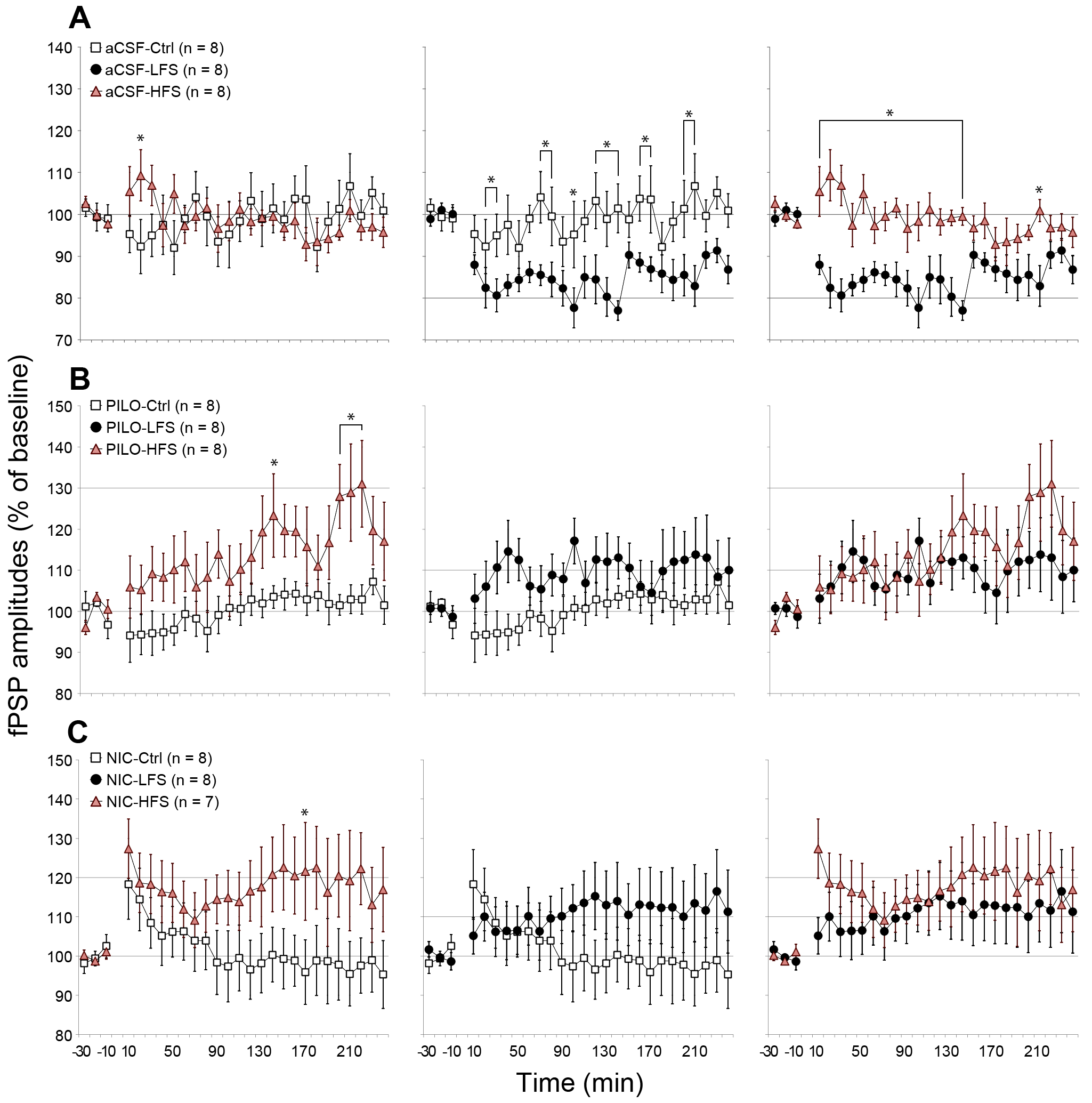

Supplement: Figure S1 — Pairwise comparisons between drug-treated groups (aCSF, PILO and NIC) in all experimental conditions (Control, LFS and HFS). Normalized amplitude of fPSPs recorded during baseline (30 min) and post-tetanization (240 min) are plotted in 10-min blocks. (A) aCSF; (B) PILO; (C) NIC. Significant differences were evaluated by two-way ANOVA with repeated measures, followed by Newman-Keuls post-hoc test. *, p<0.05. All curves correspond to data shown in figures 4–6. Data are shown as mean ± SEM. (TIF) [file pone.0047484.s001.tif]
